# Impacts of Ocean Acidification on Sediment Processes in Shallow Waters of the Arctic Ocean

**DOI:** 10.1371/journal.pone.0094068

**Published:** 2014-04-09

**Authors:** Frédéric Gazeau, Pieter van Rijswijk, Lara Pozzato, Jack J. Middelburg

**Affiliations:** 1 Sorbonne Universités, Université Pierre et Marie Curie-Paris 06, Laboratoire d'Océanographie de Villefranche, Observatoire océanologique, Villefranche/mer, France; 2 Centre National de la Recherche Scientifique-Institut National des Sciences de l'Univers, Laboratoire d'Océanographie de Villefranche, Observatoire océanologique, Villefranche/mer, France; 3 Department of Ecosystem Studies, Royal Netherlands Institute for Sea Research, Yerseke, The Netherlands; 4 Faculty of Geosciences, Utrecht University, Utrecht, The Netherlands; Institute of Marine Research, Norway

## Abstract

Despite the important roles of shallow-water sediments in global biogeochemical cycling, the effects of ocean acidification on sedimentary processes have received relatively little attention. As high-latitude cold waters can absorb more CO_2_ and usually have a lower buffering capacity than warmer waters, acidification rates in these areas are faster than those in sub-tropical regions. The present study investigates the effects of ocean acidification on sediment composition, processes and sediment-water fluxes in an Arctic coastal system. Undisturbed sediment cores, exempt of large dwelling organisms, were collected, incubated for a period of 14 days, and subject to a gradient of *p*CO_2_ covering the range of values projected for the end of the century. On five occasions during the experimental period, the sediment cores were isolated for flux measurements (oxygen, alkalinity, dissolved inorganic carbon, ammonium, nitrate, nitrite, phosphate and silicate). At the end of the experimental period, denitrification rates were measured and sediment samples were taken at several depth intervals for solid-phase analyses. Most of the parameters and processes (i.e. mineralization, denitrification) investigated showed no relationship with the overlying seawater pH, suggesting that ocean acidification will have limited impacts on the microbial activity and associated sediment-water fluxes on Arctic shelves, in the absence of active bio-irrigating organisms. Only following a pH decrease of 1 pH unit, not foreseen in the coming 300 years, significant enhancements of calcium carbonate dissolution and anammox rates were observed. Longer-term experiments on different sediment types are still required to confirm the limited impact of ocean acidification on shallow Arctic sediment processes as observed in this study.

## Introduction

Because about one third of anthropogenic CO_2_ emissions (from fossil fuel, cement production and land-use changes) has been stored in the oceans since the industrial revolution [1], surface seawater pH has already declined by ∼0.1 unit compared with pre-industrial values [2], [3] and it is projected to decrease by 0.36 unit by the end of the century [3]. Decreasing pH levels are expected to have profound impacts on the physiology and metabolism of marine organisms through a disruption of intercellular transport mechanisms [4]. Moreover, this pH decrease will modify the equilibrium between the different forms of dissolved inorganic carbon (*C*_T_) in seawater with an increase of the proportion of both CO_2_ and bicarbonate (HCO_3_^−^) at the expense of carbonate ions (CO_3_^2−^).

While it has been shown that some photosynthetic organisms will benefit from elevated CO_2_ conditions [5], most calcareous organisms have revealed a high sensitivity to the decreasing availability of CO_3_^2−^ [6]. A decrease in CO_3_^2−^ concentration leads to a reduction in the level of calcium carbonate saturation (Ω) of seawater. While it appears that most of the ocean surface will stay supersaturated with respect to all CaCO_3_ forms, high-latitude cold waters that are naturally more corrosive to CaCO_3_ than warm waters, will absorb more CO_2_ and become undersaturated with respect to high-magnesian calcite and aragonite, the most soluble forms of CaCO_3_, in few decades [7]. Acidification rates in the Arctic Ocean have shown to be faster that in sub-tropical regions [8] with some areas, as in the Canada basin and the Chukchi Sea, already experiencing undersaturated conditions [9], [10] due to a combination of sea-ice melting and anthropogenic CO_2_ penetration. These effects of freshening and increased carbon uptake in response to sea-ice retreat due to global change make Arctic Ocean surface waters the area in the world that will experience the largest pH declines in the coming decades [11]. Moreover, river runoff to Arctic shelves causes a significant decrease of alkalinity and calcium ions, and therefore lowers saturation states with respect to aragonite and calcite [12].

Shallow-water sediments play important roles in the global carbonate cycle as they represent a large reservoir of CaCO_3_ that can react to the decreasing saturation state of seawater, releasing alkalinity to the overlying water column. This dissolution of sedimentary carbonates has certainly played a significant role in past variations of atmospheric partial pressure of CO_2_ (*p*CO_2_) for instance during glacial/interglacial transitions [13]. As the rate of this reaction is both kinetically and physically limited and as the amount of CaCO_3_ is not large enough [14], it cannot compensate for the actual very fast increase of atmospheric CO_2_ [15], [16]. However, it has been suggested that dissolution of metastable carbonate phases could locally buffer changes in carbonate saturation in coral reef areas [17] for both muddy/fine grained [18], [19] and permeable sediments [20]–[22]. As high-latitudes sediments represent a much smaller CaCO_3_ reservoir than tropical areas, it is very likely that they will not have the capacity for locally buffering anthropogenic increases in CO_2_ [23].

Shallow-water sediments are also significant contributors to many biogeochemical processes in the ocean, for instance providing a large proportion of the nutrients required for primary production via organic matter degradation. Indeed, about a quarter of the organic matter that is exported from the surface of the ocean sinks onto the sediment where 90% of this organic matter is remineralized via diverse oxic or anoxic pathways [24]. Sediments are also important contributors of oceanic organic matter production via photo- or chemo-synthesis [25], [26]. Recently, several studies have discussed the role of shallow sediments in releasing alkalinity and therefore acting as a negative feedback to atmospheric CO_2_ increase, at least on the regional scale [27]–[29]. Alkalinity generating anaerobic processes (e.g. denitrification, sulfate reduction etc…) appear as important as calcium carbonate dissolution in buffering anthropogenic CO_2_ on the global scale [29]. However, despite these important roles of shallow water sediments in global biogeochemical cycling, the effects of ocean acidification on sedimentary processes have received relatively little attention. For instance, while water-column nitrification (the microbial process of ammonia oxidation to nitrite and nitrate) has been shown, through several studies, to be inhibited at low pH [30]–[32], only one study so far focused on ocean acidification effects on nitrification within sediments, actually showing a clear resilience of nitrifying microbial communities to this perturbation [32]. In contrast, several recent studies have demonstrated some indirect modifications of shallow sediment-water nutrient fluxes due to ocean acidification impacts on macro-organisms responsible for bioturbation and bioirrigation activities in the sediment [33]–[37]. Indeed, these organisms are known to enhance nutrient fluxes by increasing the penetration of oxygen (ventilation activity) and nutrients within the sediment and increasing the total sediment-water interface [38]. For instance, Dashfield et al. [36] have shown that within-sediment pH profiles and nematode community structure were significantly affected by a decrease of ∼0.5 pH unit through a modification of the activity of burrowing sea urchins.

The present study contributes to the assessment of the future role of shallow-water sediments to global carbon and nutrient cycling by investigating the effects of ocean acidification on sediment composition, processes and sediment-water fluxes in a coastal Arctic fjord. Undisturbed sediment cores were collected, incubated for a period of 14 days, and subject to a gradient of *p*CO_2_ covering the range of values projected for the end of the century (IPCC, 2007). While the present manuscript reports on sediment solid-phase composition and sediment-water fluxes, a companion paper provides information on the evolution of sediment microbial community composition [39].

## Materials and Methods

**Figure 1:**
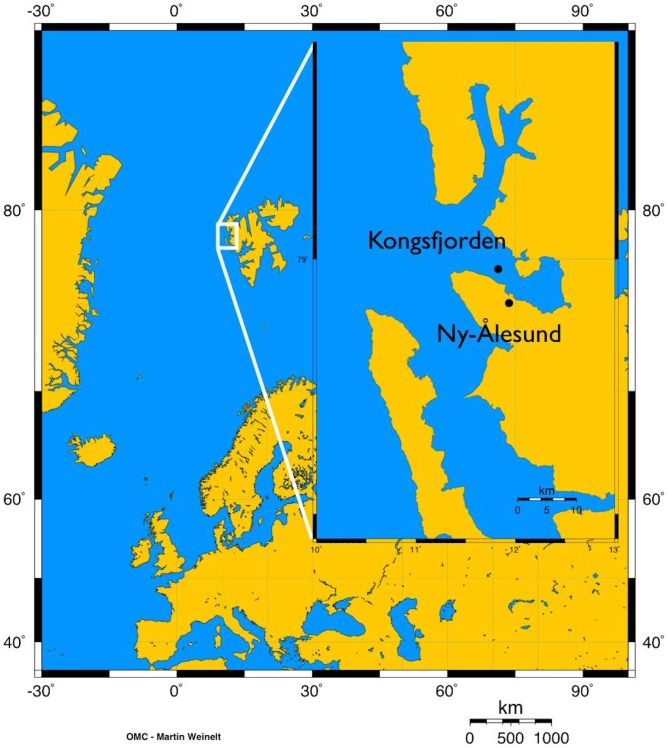
Location of the sampling area and of the experimental set-up. Sediment cores were collected near the harbour of Kongsfjorden and experiments were carried out at the marine laboratory in Ny-Ålesund (78°55′N, 11°56′E).

**Figure 2:**
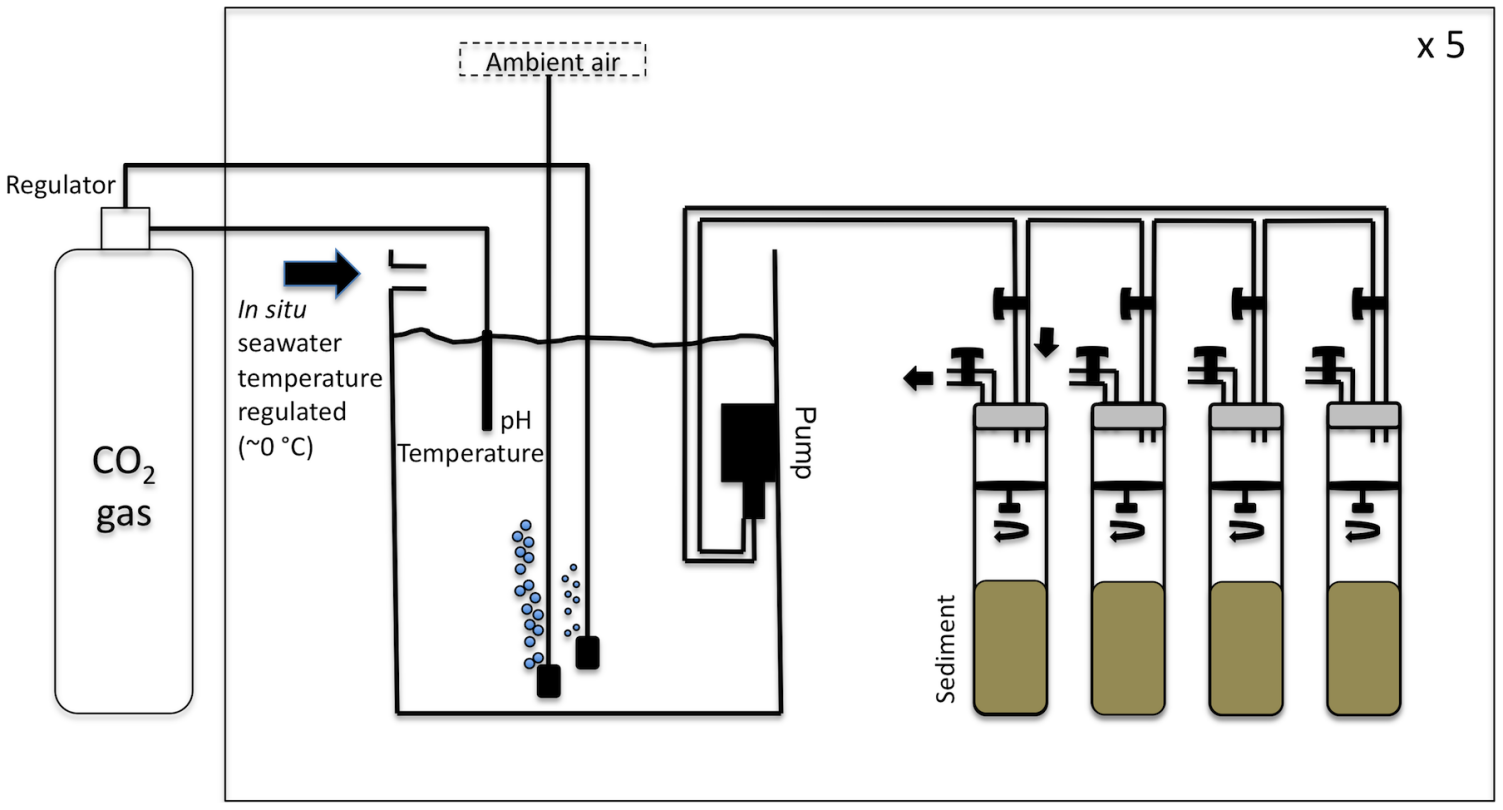
Experimental set-up used in this study. See text for details.

Sediment cores (n = 20, h = 48 cm, d = 10 cm) were collected by scuba divers at 4 m depth from an area of moderately sorted sandy mud, close to the harbour in Kongsfjorden, Svalbard (Fig. 1) on May, 10^th^ 2009 and immediately transported to the Kings Bay Marine Laboratory in Ny-Ålesund. No specific permissions were required for sampling at this location. Sediment was ∼70% silt and ∼20% very fine sand, and contained ∼2.5% and ∼2.6% of organic and inorganic carbon, respectively. In the laboratory, during 14 days, the sediment cores were kept in darkness and continuously supplied with temperature-regulated *in situ* seawater (∼0°C), pumped at a rate of 5 ml min^−1^ from header tanks (n = 5, 200 l, Fig. 2) in which *p*CO_2_ was controlled and maintained through pure CO_2_ bubbling using flat surface, combination pH electrodes (Walchem S650CD) and an automated feedback relay system. Five different *p*CO_2_ levels were considered: *in situ* conditions (no CO_2_ bubbling; ∼317 μatm), 540, 760, 1120 and 3000 μatm. Four sediment cores were used per treatment.

This perturbation design has been considered in order to encompass the different scenarios that are projected by IPCC climate models for the end of the century (540 to over 1000 ppm depending on the considered emission scenario [40]). The 3000 μatm treatment has been used as an extreme case study.

Corresponding pH levels used for the regulation system were calculated every other day using the R package seacarb (Lavigne and Gattuso, 2010) based on total alkalinity (*A*_T_, see below) measurements and desired *p*CO_2_ levels. To ensure an optimal aeration and a better dissolution of CO_2_ in seawater, header tanks were continuously and actively bubbled with ambient air. Four cores were supplied with seawater from each header tank. pH was measured every second day in the 5 header tanks using a pH meter (Metrohm, 826 pH mobile) with a glass electrode (Metrohm, electrode plus) calibrated on the total scale using Tris/HCl and 2-aminopyridine/HCl buffer solutions with a salinity of 35.0 at the temperature of the experimental setup (Dickson et al., 2007). Simultaneously to the pH recording, 100 ml of water from the control tank were carefully sampled and filtered on GF/F for *A*_T_ measurements that were performed within two days using a potentiometric titration and a Metrohm titrator (Titrando 80). Measurements were carried out in triplicate on 25 ml samples at 21°C and *A*_T_ was calculated with a precision of 1–2 μmol kg^−1^ using a Gran function applied to the pH_T_ values ranging from 3.5 to 3.0 as described by Dickson et al. [41]. Titrations of standard seawater provided by A. G. Dickson (batch 95) yielded *A*_T_ values within 1.7 μmol kg^−1^ of the nominal value (2216.45 μmol kg^−1^; standard deviation  = 1.85 μmol kg^−1^; n = 12). All the parameters of the carbonate chemistry, including dissolved inorganic carbon (*C*_T_) concentrations were determined from pH_T_, *A*_T_, temperature and salinity using the R package seacarb (Lavigne and Gattuso, 2012).

On five occasions during the experimental period (14 days), the sediment cores were isolated during 24 h for flux measurements. Before and after incubation, seawater samples were taken for pH_T_ (see above), *A*_T_ (see above), dissolved oxygen (DO) and nutrient analyses: ammonium (NH_4_^+^), nitrate (NO_3_^−^), nitrite (NO_2_^−^), dissolved inorganic phosphorus (DIP) and dissolved silicate (Si). Nutrients were measured with an automated colorimetric method [42] with a precision better than 4% and the accuracy was verified through use of certified standards and participation in QUASIMEME robin round tests with a Z-score <0.5. DO concentrations were measured using an automated Winkler titration technique with a potentiometric end-point detection. Analyses were performed with a Metrohm redox electrode and a custom built titrator. Reagents and standardizations were similar to those described by Knap et al. [43].

At the end of the experimental period, denitrification rates were measured in each core over a 24 h incubation period using ^15^N tracers. Enriched sodium nitrate (^15^N 98%, Cambridge Isotope Laboratories Inc) was added to core overlying water to reach a final enrichment of 50 at% ^15^N for nitrate. Samples of overlying water (20 ml) were taken at 0, 4, 12 and 24 h for ^15^N-N_2_ measurements [44], which were performed using a Thermo Flash EA 1112 elemental analyzer coupled via a Conflo III interface to an Isotope Ratio Mass Spectrometer (Thermo Delta V Advantage). Denitrification rates were estimated using the following equation:
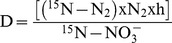
(1)


Where D is denitrification (in μmol m^−2^ d^−1^), ^15^N-N_2_ is enrichment rate of N_2_ in the overlying water with time (in atom % d^−1^), N_2_ is the N_2_ concentration in the overlying water (in μmol m^−3^) which was assumed constant, at saturation and calculated based on [45], h is the water height in the sediment cores (in m) and ^15^N-NO_3_^−^ is the atom % ^15^N-NO_3_^−^ in the overlying water. During these incubations, bio-irrigation rates were quantified using the bromide technique [46]. Sodium bromide was added in the overlying water at the start of the incubations (concentration of ∼0.9 mmol L^−1^). Bio-irrigation was estimated as the decrease of bromide concentration with time by sampling 20 ml of the overlying water at 0, 4, 12 and 24 h. Bromide was measured by reverse-phase high-performance liquid chromatography (HPLC).

After the end of the last incubations for denitrification and bio-irrigation measurements, sediment samples were taken at depth intervals of 0–0.5, 0.5–1, 1–2, 2–4 and >4 cm and freeze-dried for subsequent analyses of grain size distribution, pigment concentrations and total/organic carbon and total nitrogen content as well as isotopic compositions (^13^C) of total and organic carbon and isotopic compositions (^15^N) of organic nitrogen. Grain-size distribution was assessed with a laser diffraction technique (Malvem Mastersizer 2000). Pigments (chlorophyll *a*, *b* and *c*) were extracted from freeze-dried samples with 10 ml 90/10 acetone/water buffered with ammonium acetate (5%), and analyzed by HPLC. Measurements of total and organic carbon (C_tot_ and C_org_, respectively) and total nitrogen (N_tot_) were made using a Fisons NA-2500 elemental analyzer [47]. The carbon (total and organic) and organic nitrogen isotopic composition of the sediment was determined using a Thermo Flash EA 1112 elemental analyzer coupled via a Conflo III interface to an Isotope Ratio Mass Spectrometer (Thermo Delta V Advantage). The carbon and nitrogen isotope ratios are expressed in the delta notation δ^13^C and δ^15^N, respectively, where: δ = (R_sample_/R_standard_−1)×1000

Results are referred to Vienna PDB for C and to atmospheric nitrogen for N and expressed in units of ‰. Reproducibility of the measurements is better than 0.2 ‰ [48].

### Statistics

Principal components analyses (PCA) were used to test 1) whether the sediment cores used in this study were similar in terms of grain size distribution (5 variables, 96 samples), 2) the effects of *p*CO_2_ and depth of sampling in the sediment on the composition of the sediment with respect to the different analyzed variables (pigments, C_tot_, C_org_, N_tot_, δ^13^C_tot_, δ^13^C_org_ and δ^15^N_org_; 9 variables, 87 samples) and 3) the effects of the environmental conditions to which the sediment cores were exposed during the incubations (*p*CO_2_ and temperature) and the effects of time on sediment-water fluxes (10 variables, 90 samples). A one-way ANOVA was used to test the effects of seawater *p*CO_2_ on denitrification rates. Data were first checked to ensure they conformed to the assumptions of ANOVA (normality: Kolmogorov-Smirnov test and homoscedasticity: Bartlett test). All statistical analyses were conducted using the R software and PCA analyses were performed using the ADE4 package.

## Results

Environmental conditions in the header tanks, from which seawater was pumped and distributed to the sediment cores, are presented in Table 1. Seawater temperature averaged 0.18±0.53°C during the experimental period. Seawater pH_T_ was maintained at 8.13±0.08, 7.84±0.11, 7.71±0.21, 7.57±0.08 and 7.17±0.06 in the control, 540, 760, 1120 and 3000 μatm header tanks respectively. Variations were the highest in the 760 μatm tank due to a technical problem that lasted for few hours and during which seawater pH_T_ dropped to 7.07. *C*_T_ concentration increased from 2126±28 to 2454±31 μmol kg^−1^ between the lowest and highest *p*CO_2_ treatment. Seawater was, on average, only supersaturated with respect to aragonite in the control treatment while in the other tanks Ω_aragonite_ decreased from 0.9±0.2 to 0.2±0.0 between the 540 and the 3000 μatm treatments. Seawater saturation with respect to calcite was below 1 for the two highest *p*CO_2_ treatments, with the lowest value for the 3000 μatm tank at 0.33±0.04, and above 1 for the 3 other treatments.

**Table 1:** Seawater temperature and carbonate chemistry controlled in the header tanks during the course of the experiment (∼14 days; n = 7).

		Tank 1	Tank 2	Tank 3	Tank 4	Tank 5
Target *p*CO_2_ (μatm)		Control	540	760	1120	3000
Water temperature (°C)	Mean	0.44	0.24	0.13	0.03	0.08
	SD	0.70	0.78	0.31	0.31	0.27
	Min	−0.40	−0.50	−0.50	−0.50	−0.40
	Max	2.00	2.10	0.50	0.50	0.50
pH_T_	Mean	8.13	7.84	7.71	7.57	7.17
	SD	0.08	0.11	0.21	0.08	0.06
	Min	8.01	7.65	7.07	7.44	7.07
	Max	8.30	8.14	7.94	7.71	7.26
*C*_T_ (μmol kg^−1^)	Mean	2126.1	2223.3	2267.7	2304.3	2454.3
	SD	27.9	32.9	74.1	24.8	30.7
	Min	2062.4	2134.2	2199.8	2263.6	2405.3
	Max	2165.3	2288.3	2515.4	2345.3	2510.3
*p*CO_2_ (μatm)	Mean	317.2	658.5	1031.2	1255.8	3169.4
	SD	60.3	162.3	815.3	235.3	437.7
	Min	200.2	306.0	507.2	874.4	2539.7
	Max	422.3	1024.3	3959.6	1674.2	3981.6
Ω_aragonite_	Mean	1.68	0.93	0.72	0.50	0.21
	SD	0.24	0.22	0.23	0.09	0.03
	Min	1.39	0.59	0.16	0.38	0.16
	Max	2.25	1.66	1.12	0.69	0.25
Ω_calcite_	Mean	2.67	1.48	1.15	0.80	0.33
	SD	0.38	0.36	0.37	0.15	0.04
	Min	2.22	0.94	0.25	0.60	0.25
	Max	3.58	2.65	1.78	1.10	0.39

Salinity was constant throughout the experiment at 34.5. Measured pH in the total scale (pH_T_), dissolved inorganic carbon (*C*_T_) as well as calculated *p*CO_2_ and saturation states with respect to aragonite (Ω_aragonite_) and calcite (Ω_calcite_), are presented as mean, standard deviation (SD), minimal (Min) and maximal (Max) values.

**Figure 3:**
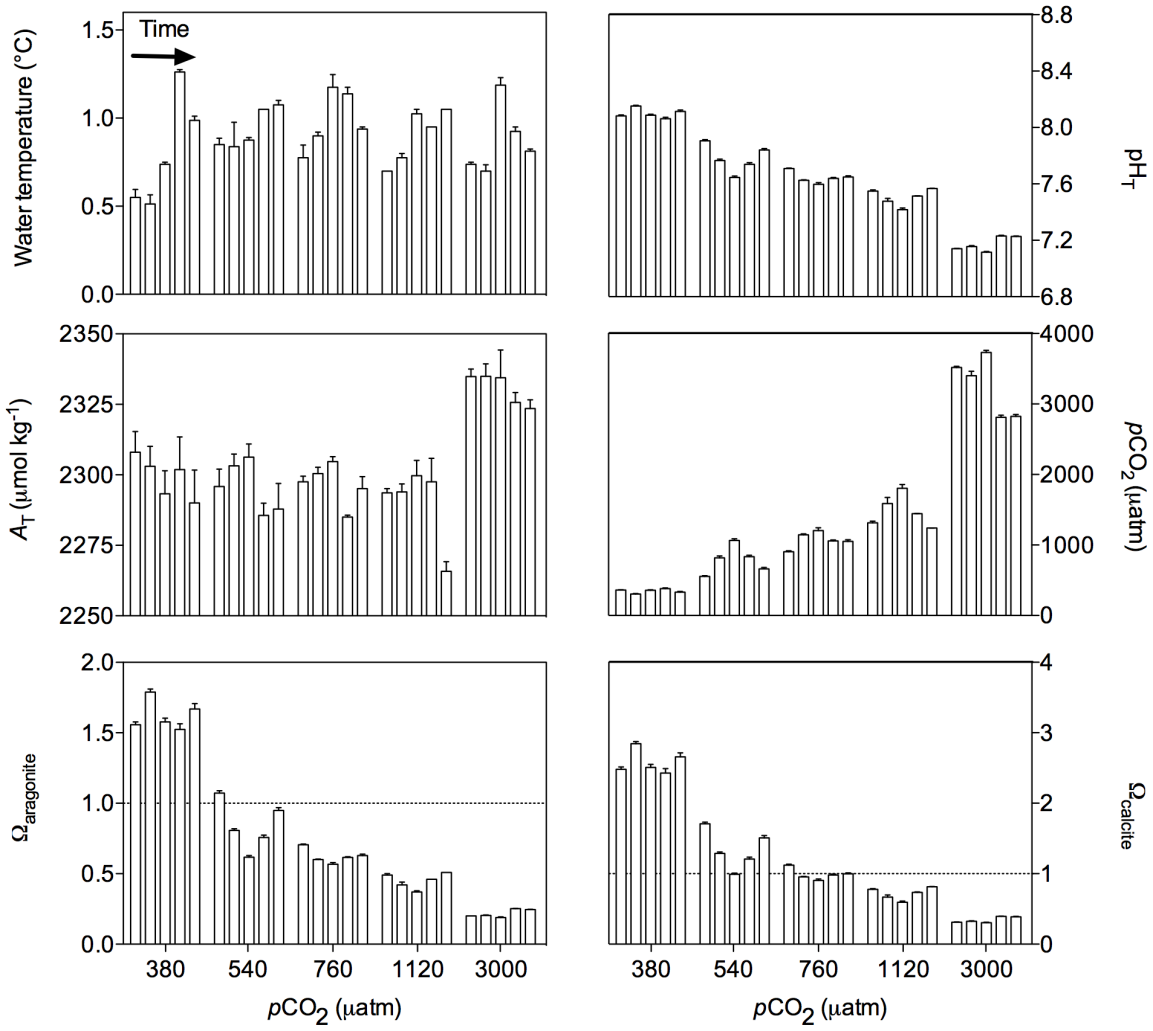
Experimental conditions (average between initial and final sampling) at which the sediment cores were exposed during the 24 h incubations (n = 5). :pH_T_: pH on the total scale, *A*_T_: total alkalinity, Ω_aragonite_ and Ω_calcite_: saturation state of the overlying seawater with respect to, respectively, aragonite and calcite. Error bars represent the standard deviation.

Environmental conditions during the 24 h incubations (n = 5) are presented in Fig. 3. Data represent average values between initial and final sampling for the four cores used per treatment (± SD). For each *p*CO_2_ treatment, pH_T_ was relatively constant during the incubations and throughout the experimental period with the highest variation observed for the 540 μatm condition. Higher *A*_T_ levels were observed for the 3000 μatm treatment due to the significant release of *A*_T_ during the incubations. During the incubations, seawater was undersaturated with respect to aragonite in all cores except the ones from the control treatment. Sediment cores from the control and 540 μatm treatments were exposed to supersaturated conditions with respect to calcite (mean: 2.6±0.2 and 1.3±0.3 respectively) while those from the 1120 and 3000 μatm treatments were maintained below saturation (mean: 0.7±0.1 and 0.3±0.0 respectively) and those from the 760 μatm treatment were close to saturation (mean: 1.0±0.1).

The PCA on the sediment grain size distribution (data not shown) revealed some discrepancies between the different sediment cores used in this study (n = 20). Cores 13 and 14 that were exposed to 1120 μatm had a much larger fraction of medium and fine sand grains than the others. Also, the deepest depth-layer of core 20 exposed to 3000 μatm contained a much larger fraction of coarse sand grains. As all the other cores appeared to share the same grain size characteristics, it has been decided to remove from the subsequent analysis cores 13 and 14 as well as the deepest depth-layer of core 20. Averaged (±SD) sediment grain size distributions in the remaining cores for the different depth layers are shown in Table 2. The sediment cores had a thick coal layer at the bottom.

**Table 2:** Sediment size distribution (%) in the different depth layers, expressed as an average (SD) value between the different cores used in the experiment.

Depth layer (cm)	Silt	Very fine sand	Fine sand	Medium sand	Coarse sand
	<62.5 μm	62.5–125 μm	125–250 μm	250–500 μm	500–1000 μm
0–0.5	65.7 (3.0)	21.3 (1.6)	8.0 (0.9)	3.3 (0.5)	2.0 (0.6)
0.5–1	64.4 (2.0)	23.0 (1.2)	8.3 (0.9)	2.9 (0.5)	1.7 (0.7)
1–2	66.5 (2.9)	22.2 (2.0)	7.6 (1.1)	2.5 (0.4)	1.5 (0.8)
2–4	72.9 (3.3)	19.2 (2.6)	5.8 (0.8)	1.6 (0.2)	0.8 (0.3)
>4	77.1 (3.0)	14.3 (1.5)	4.2 (0.5)	1.6 (0.4)	3.0 (2.0)

Cores 13 and 14 (1120 μatm treatment) as well as the layer deeper than 4 cm of core 20 (3000 μatm treatment) have not been considered in this analysis and in the subsequent ones as they have been showed to greatly differ from the other samples (See text for details).

**Figure 4:**
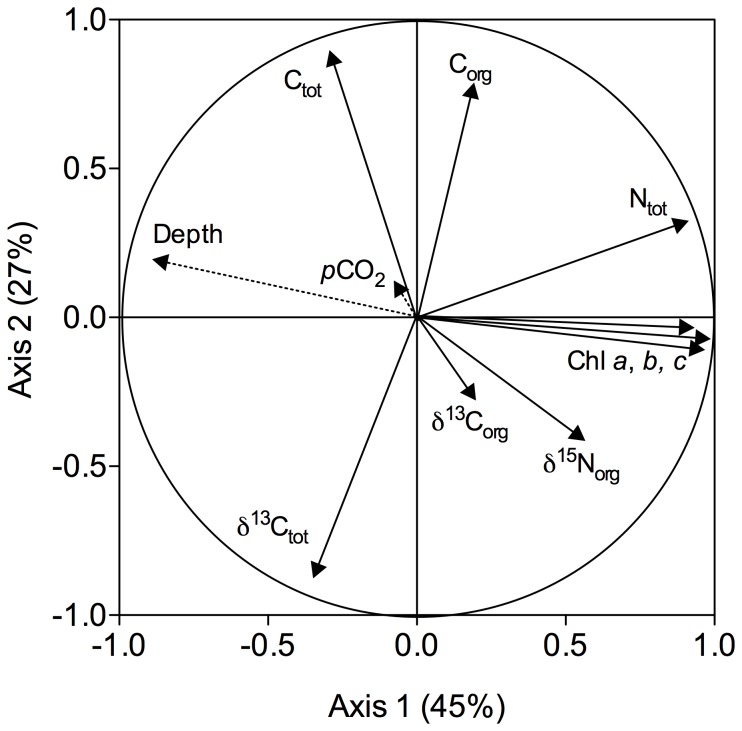
Correlation circle from the principal component analysis (PCA) applied on the composition of the sediment with respect to carbon and nitrogen (C_tot_ and N_tot_), to organic carbon (C_org_), to the isotopic composition of C_tot_ and C_org_ (δ^13^C_tot_ and δ^13^C_org_) and organic nitrogen (δ^15^N_org_) as well as to the pigments concentrations (chlorophyll *a*, *b* and *c*). Seawater targeted *p*CO_2_ ( μatm) and the depth of sampling (cm) in the sediment have been added as supplementary variables. Axis 1 and axis 2 explained, respectively, 45 and 27% of the overall variance in the data.

The correlation circle of the PCA performed on the composition of the sediment with respect to carbon and nitrogen (C_tot_ and N_tot_), organic carbon (C_org_), the isotopic composition of C_tot_ and C_org_ (δ^13^C_tot_ and δ^13^C_org_) and organic nitrogen (δ^15^N_org_), as well as the pigments concentrations (chlorophyll *a*, *b* and *c*) is presented in Fig. 4 for the two axis explaining most of the variance in the data (axis 1: 45% and axis 2: 27%). Seawater targeted *p*CO_2_ (μatm) and the depth of sampling (cm) in the sediment have been added as supplementary variables. Data used for this analysis and the complete correlation matrix are presented in Tables S1 and S2, respectively. This analysis showed no significant effect of seawater *p*CO_2_ on any of these parameters and *p*CO_2_ was not correlated to the composite axes 1 and 2. Most of the variance in the dataset was explained by the depth at which parameters were measured in the sediment with significant decreases of N_tot_ as well as of Chl *a*, *b* and *c* with depth, because of active diagenesis.

**Figure 5:**
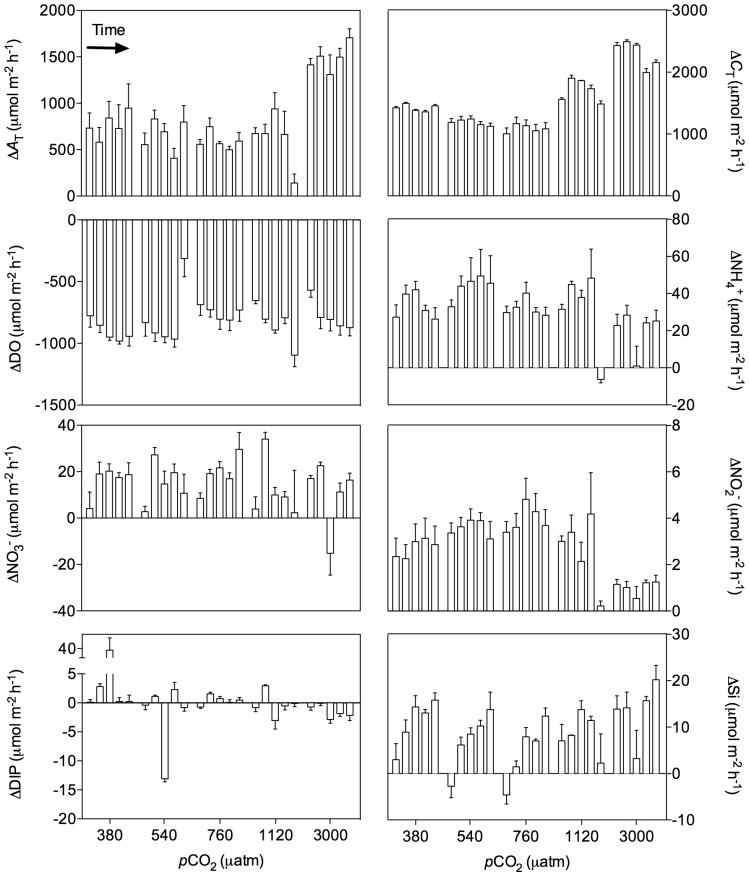
Sediment-water fluxes of total alkalinity (Δ*A*_T_), dissolved inorganic carbon (Δ*C*_T_), ammonium (ΔNH_4_^+^), nitrate (ΔNO_3_^−^), nitrite (ΔNO_2_^−^), dissolved inorganic phosphorus (ΔDIP) and silicate (ΔSi) observed during the 24 h incubations (n = 5). Positive rates represent fluxes from the sediment to the overlying water.

**Figure 6:**
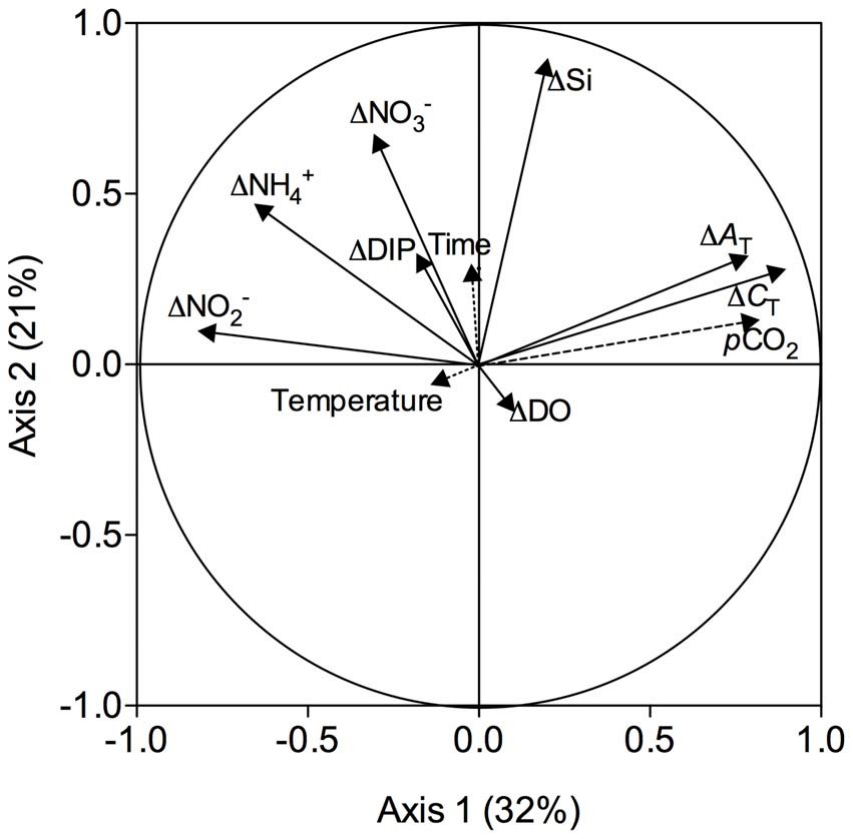
Correlation circle from the principal component analysis (PCA) applied on sediment-water fluxes of total alkalinity (*A*_T_), dissolved inorganic carbon (*C*_T_), ammonium (NH_4_^+^), nitrate (NO_3_^−^), nitrite (NO_2_^−^), dissolved inorganic phosphorus (DIP) and silicate (Si). Seawater temperature (°C) and *p*CO_2_ (μatm) at which sediment cores were exposed during the incubations (average between initial and final sampling) as well as the time elapsed since the start of the experiment (in days) have been added as supplementary variables. Axis 1 and axis 2 explained, respectively, 32 and 21% of the overall variance in the data.

Results from the flux measurements during the 24 h incubations are shown in Fig. 5. At all *p*CO_2_ levels and for all incubations, the sediment was a source of *A*_T_, *C*_T_ and NO_2_^−^ and a sink of DO for the overlying water. The sediment was also a source of NH_4_^+^ and NO_3_^−^ for the overlying water, except for one incubation at 1120 μatm and at 3000 μatm for NH_4_^+^ and NO_3_^−^ respectively. The sediment was most of the time a source of Si except at the start of the experiment at 540 and 760 μatm. DIP fluxes were quite variable with a maximal efflux rate of 114.5 and a maximal influx rate of −14.6 μmol m^−2^ h^−1^. The correlation circle of the PCA ran on these fluxes is presented in Fig. 6 for the two axis explaining most of the variance in the data (axis 1: 32% and axis 2: 21%). Seawater temperature and *p*CO_2_ at which sediment cores were exposed during the 24 h incubations (average between initial and final sampling) as well as the time elapsed since the start of the experiment (in days) have been added as supplementary variables. The complete correlation matrix is presented in Table S3. This analysis shows that *A*_T_, *C*_T_, NH_4_^+^ and NO_2_^−^ fluxes were correlated with *p*CO_2_ (p<0.01). *A*_T_ and *C*_T_ fluxes were positively correlated while NH_4_^+^ and NO_2_^−^ fluxes were negatively correlated. The second axis is mainly explained by the variance in Si fluxes that appeared to be significantly correlated with time (p<0.001). Indeed, as shown in Fig. 4, Si fluxes generally increased during the experimental period. Temperature did not have significant effects on any of the observed fluxes. Visual inspections of the evolution of *A*_T_, *C*_T_, NH_4_^+^ and NO_2_^−^ presented in Fig. 5 suggest that the 3000 μatm treatment is dominant in the observed significant correlations between these fluxes and *p*CO_2_ levels with larger *C*_T_ and *A*_T_ fluxes and relatively lower NH_4_^+^ and NO_2_^−^ fluxes compared to the ones measured at lower *p*CO_2_ levels.

**Figure 7:**
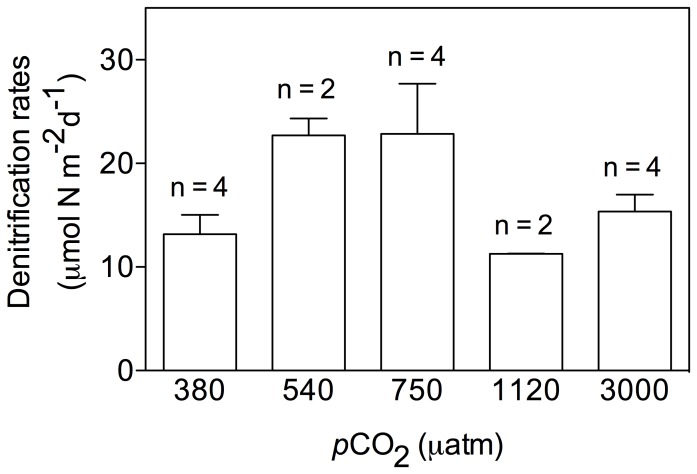
Denitrification rates measured over the 24*p*CO_2_ levels considered in the present study.

Denitrification rates estimated based on ^15^N-NO_3_^−^ labeling are shown in Fig. 7. Increases in ^15^N-N_2_ were significant and linear over 24 h except for 2 cores from the 540 μatm treatment which, most certainly due to technical problems, did not show any denitrification activities and were therefore excluded from the analysis. Denitrification rates ranged between 11 and 35 μmol N m^−2^ d^−1^ and did not significantly differ (one-way ANOVA, p = 0.1) between the different *p*CO_2_ treatments. During these last incubations, there was no bio-irrigation activity as no significant decrease of bromide has been detected after the 24 h incubation in any of the treatments (data not shown).

## Discussion

Most of the parameters and processes investigated within this study showed no relationship with the overlying seawater pH. Solid-phase concentrations did not change over the 14 day-time period of the experiment. This does not appear as a surprise as changes in sediment solid-phases are usually difficult to detect over short time-scales and effects are stronger reflected in pore-water concentrations and sediment-water exchange rates [49]. The 14-day duration of our experiments may also have limited the impact on slowly reacting pools at depth in the sediments. The increase of silicate fluxes with time (Fig. 5) likely reflects a transient response of biogenic silica dissolution or acidification induced enhanced chemical weathering.

*C*_T_ fluxes were the lowest at ∼800 μatm and increased below and above this level. This pattern is due to the decrease in the *C*_T_ gradient between water and sediment up to 800 μatm and potentially an increase in carbonate dissolution rates above this level. Interestingly, enhanced sediment carbonate dissolution (resulting in an increased *A*_T_ release from the sediment) only started at the lowest pH conditions (*p*CO_2_ ∼3000 μatm) when strong undersaturations with respect to both aragonite and calcite occurred (Ω_aragonite_ = 0.21, Ω_calcite_ = 0.33). At 1120 μatm, the overlying water was also corrosive to calcite, but did not seem to induce enhanced dissolution rates. Unfortunately, as no pH, *C*_T_ and/or *A*_T_ sediment profiles were acquired during this study, it is impossible to investigate the capacity of the sediment to buffer pH changes in pore-waters.

It has to be noted that variations in the overlying water *A*_T_ during the 24 h incubations are not only due to carbonate dissolution. Remineralization of particulate organic matter and subsequent nutrient release affect *A*_T_ depending on the form of reactive nitrogen produced [50], [51]. A release of 1 mole of ammonia or nitrate leads to an increase or decrease, respectively, of *A*_T_ by 1 mole. Nitrification is also known to significantly impact *A*_T_ (decrease of 2 moles per moles of NO_3_^−^ produced) while nitrification/denitrification coupling has no effect on *A*_T_ fluxes. Release of phosphate during remineralization leads to a corresponding decrease of *A*_T_. During the present study, *A*_T_ release from the sediment was most likely due to oxic mineralization induced carbonate dissolution, as C_T_ and DO fluxes were by 1–2 orders of magnitude higher than rates of nutrient release/consumption. Since oxygen uptake, and by inference oxic mineralization was not impacted by decreases in pH, the enhanced dissolution rates as observed at the lowest pH levels were most likely directly linked to pH of the overlying water that penetrated to a certain extent in the sediment. It must be stressed that sulfate reduction rates have not been measured during the present study. Sulfate reduction may indeed increase alkalinity in the sediments, but most of the reduced products generated by anaerobic mineralization are re-oxidized leading to a decrease in *A*_T_ via proton production, hence balancing the initial *A*_T_ production [27], [51]. It is very unlikely that an alteration of this process may have been responsible for the increased *A*_T_ fluxes observed at low pH. *A*_T_ fluxes measured during this study are significantly lower that rates measured for tropical carbonate muds [19] but in the range on what has been measured in carbonate sands [21] although the carbonate content of the sediment in the present study were much lower (21% CaCO_3_). Values found in this study are in the same range that what has been reported for the Arctic in general (14±8%CaCO_3_ [52]).

This is, to the best of our knowledge, the first report on the effect of ocean acidification on sediment oxygen fluxes in the Arctic. It must be stressed that, for logistic reasons, our experiment was conducted in darkness, and that the potential effects of increased CO_2_ on autotrophic processes were not taken into account. During this study, no significant alteration of the oxygen consumption in the overlying water following pH decreases has been shown. Oxygen is used during both organic matter mineralization and re-oxidation processes such as nitrification, the biological oxidation of ammonia into nitrite followed by the oxidation of this nitrite into nitrate [53]. Significant effects of seawater acidification on pelagic nitrification rates have been shown by Huesemann et al. [31], Beman et al. [30] and Kitidis et al. [32], based on both laboratory and field studies. As the NH_3_/NH_4_^+^ equilibrium is pH sensitive and in favor of NH_4_^+^ at lower pH, this has been formulated as an explanation for observed decreasing nitrification rates in these studies. Only one study so far focused on the effect of ocean acidification on sediment nitrification rates. In contrast to what has been found for pelagic nitrification, Kitidis et al. [32] showed no effect of a decrease in pH (up to −0.6) on benthic nitrification rates. Based on ammonium, nitrite and oxygen sediment-water fluxes as well as on denitrification rates, it seems unlikely that, in our study, nitrification rates have been impacted by lowered pH as most of the denitrified nitrate came from nitrification in the sediment. A modification of the nitrification activity would have undoubtedly had effects on coupled denitrification rates, which was clearly not the case in our study. The decrease in ammonium and nitrite release, observed only at the lowest experimental pH (i.e. 3000 μatm), could be attributed to an enhanced anammox activity, the anaerobic biological process converting nitrite and ammonium directly into dinitrogen gas [54]. In a companion paper [39], an increase in the abundance of Planctonomycete-specific 16S rRNA, indicative of anammox bacteria, has been shown and several studies already suggested that this process will be favored in a higher CO_2_ ocean [34]. Interestingly, Tait et al. [39] also showed that effect of increasing *p*CO_2_ was significant in terms of bacterial community composition at the intermediate levels of 540 and 760 μatm, with reduced abundance of bacterial amoA transcript abundance (aerobic oxidizers) at 760 μatm and above. However, the abundance of archaeal amoA transcripts did not change within these treatments and actually increased at 3000 μatm, suggesting that bacterial ammonia oxidisers may be more vulnerable to increased CO_2_ than archaeal ammonia oxidisers. The lack of response of nitrogen flux measurements at the intermediate CO_2_ levels may have been due to continued archaeal amoA activity.

Previous studies focusing on the effects of ocean acidification on sediment-water fluxes reported stronger impacts than the ones presented here [33]–[37]. First of all, these experiments considered usually much lower pH levels than the ones projected for the end of the century. Moreover, the observed impacts were, in most cases, indirect and attributed to significant effects of ocean acidification on bioturbating and bio-irrigating organisms [33]–[37]. As mentioned previously, the sediment used in the present study was exempt of these organisms and transport processes were dominated by molecular diffusion. Although enhanced dissolution rates at the lowest pH levels showed that pH of overlying water had an impact on the sediment, the absence of sediment pH microprofiles during this study does not allow assessing the penetration of ocean acidification perturbation, after 14 days of exposure, into the sediment.

The absence of bioturbating organisms is a common feature of the sediments in the inner fjord where samples were taken from [55], but large abundances of bioturbating organisms can be found in the central and outer fjord. It was initially planned to sample the sediment cores in the central fjord but a thick ice cover blocked access to the majority of the fjord adjacent to Ny-Ålesund and made collection of samples and sediment cores extremely difficult. Net sediment-water fluxes of nitrogen and oxygen have been measured from sediments sampled in the same fjord but in deeper areas [56]. While oxygen uptakes were 6 times lower than the ones measured in the present study (4 vs. ∼24 mmol m^−2^ d^−1^), their sediment acted as a sink of NH_4_^+^ for the overlying water, as opposed to the constant net efflux observed in our study. In contrast, NO_3_^−^ efflux appeared very similar between the two studies with values around 300 μmol m^−2^ d^−1^. As already mentioned, the sediments used in the present study were much shallower. As such, extrapolations based on our results should be done with care.

Based on the present results, we can conclude that ocean acidification, under the conditions of our experiment, will have limited impacts on sediment carbonate dissolution, on the microbial activity (e.g. oxic and anoxic mineralization, nitrification) and associated sediment-water fluxes on Arctic shelves. The potential effects that have been shown regarding carbonate dissolution and the anammox activity occurred at the lowest experimental pH (∼3000 μatm), a level that is not foreseen within the next 100 years. However, as discussed previously, the sediment used in this study was exempt of bio-irrigating organisms and the penetration of low pH water inside the sediment might have been limited following an exposure time of 14 days. Longer-term experiments on different sediment types and under different light conditions (different seasons) are still required to confirm the limited impact of ocean acidification on shallow Arctic sediment processes as observed in this study.

## Supporting Information

Table S1 **Composition of all sediment cores (at several depths, in cm) after a 14 days exposure at various partial pressure of CO_2_ levels (** ***p*** **CO_2_ in μatm, 4 cores per treatment).** The content in carbon and nitrogen (C_tot_ and N_tot_ in %), organic carbon (C_org_ in %), the isotopic composition of C_tot_ and C_org_ (δ^13^C_tot_ and δ^13^C_org_ in ‰) and organic nitrogen (δ^15^N_org_ in ‰), as well as the pigment concentrations (chlorophyll *a*, *b* and *c* in μg g^−1^) are presented.
(DOCX)

Table S2 **Correlation matrix associated with the PCA performed on the composition of the sediment at the end of the experiment (14 days) with respect to carbon and nitrogen (C_tot_ and N_tot_), organic carbon (C_org_), the isotopic composition of C_tot_ and C_org_ (δ^13^C_tot_ and δ^13^C_org_) and organic nitrogen (δ^15^N_org_), as well as the pigments concentrations (chlorophyll** ***a*** **,** ***b*** **and** ***c*** **).** Seawater targeted partial pressure of CO_2_ (*p*CO_2_ in μatm) and the depth of sampling (cm) in the sediment have been added as supplementary variables. The first two axes of the PCA represented 45 and 27% of the variance, respectively. All correlation values were tested for significance (Pearson correlation test performed on R, package Psych). Values in bold are associated with a *p* value below 0.01.
(DOCX)

Table S3 **Correlation matrix associated with the PCA performed on the sediment-water fluxes of total alkalinity (Δ** ***A*** **_T_), dissolved inorganic carbon (Δ** ***C*** **_T_), dissolved oxygen (ΔDO), ammonium (ΔNH_4_^+^), nitrite (ΔNO_2_^−^), nitrate (ΔNO_3_^−^), dissolved inorganic phosphate (ΔDIP) and silicate (ΔSi).** Seawater temperature (Temp) and partial pressure of CO_2_ (*p*CO_2_) at which sediment cores were exposed during the 24 h incubations (average between initial and final sampling) as well as the time elapsed since the start of the experiment (in days) have been added as supplementary variables. The first two axes of the PCA represented 32 and 21% of the variance, respectively. All correlation values were tested for significance (Pearson correlation test performed using the R software, package Psych). Values in bold are associated with a *p* value below 0.01.
(DOCX)
