# Supplementary material for: Impacts of Ocean Acidification on Sediment Processes in Shallow Waters of the Arctic Ocean
Source: PLoS One. 2014 Apr 9;9(4):e94068. doi: 10.1371/journal.pone.0094068 (PMC3981760; doi:10.1371/journal.pone.0094068)
Supplement: Table S1 — Composition of all sediment cores (at several depths, in cm) after a 14 days exposure at various partial pressure of CO2 levels ( p CO2 in μatm, 4 cores per treatment). The content in carbon and nitrogen (Ctot and Ntot in %), organic carbon (Corg in %), the isotopic composition of Ctot and Corg (δ13Ctot and δ13Corg in ‰) and organic nitrogen (δ15Norg in ‰), as well as the pigment concentrations (chlorophyll a, b and c in μg g−1) are presented. (DOCX) [file pone.0094068.s001.docx]

| Core # | *p*CO_2_ | Depth | Chl *a* | Chl *b* | Chl *c* | C_tot_ | C_org_ | N_tot_ | δ^13^C_tot_ | δ^13^C_org_ | δ^15^N_org_ |
| --- | --- | --- | --- | --- | --- | --- | --- | --- | --- | --- | --- |
| 1 | 380 | 0.25 | 23.1 | 5.4 | 1.7 | 4.8 | 2.5 | 0.2 | -13.2 | -24.2 | 33.7 |
| 1 | 380 | 0.75 | 21.2 | 4.8 | 1.3 | 4.8 | 2.5 | 0.2 | -12.0 | -24.1 | 12.7 |
| 1 | 380 | 1.5 | 14.7 | 1.7 | 1.0 | 4.9 | 2.5 | 0.1 | -11.4 | -24.2 | 7.1 |
| 1 | 380 | 3 | 8.2 | 0.7 | 0.5 | 5.2 | 2.2 | 0.1 | -10.8 | -24.4 | 6.4 |
| 1 | 380 | 4 | 3.4 | 0.2 | 0.2 | 5.5 | 3.3 | 0.1 | -12.4 | -24.9 | 5.7 |
| 2 | 380 | 0.25 | 14.6 | 2.0 | 1.1 | 4.2 | 2.4 | 0.2 | -11.7 | -24.4 | 29.6 |
| 2 | 380 | 0.75 | 20.0 | 3.0 | 1.6 | 5.2 | 2.9 | 0.2 | -12.0 | -24.4 | 9.7 |
| 2 | 380 | 1.5 | 13.4 | 1.8 | 1.0 | 5.2 | 2.7 | 0.2 | -12.1 | -24.5 | 7.4 |
| 2 | 380 | 3 | 7.0 | 0.5 | 0.5 | 5.0 | 2.4 | 0.1 | -9.5 | -24.5 | 5.7 |
| 2 | 380 | 4 | 2.4 | 0.3 | 0.2 | 4.9 | 2.1 | 0.1 | -11.3 | -24.6 | 7.9 |
| 3 | 380 | 0.25 | 29.7 | 6.8 | 2.2 | 4.3 | 2.5 | 0.2 | -12.0 | -23.9 | 21.9 |
| 3 | 380 | 0.75 | 23.0 | 2.8 | 1.5 | 5.3 | 2.8 | 0.2 | -13.2 | -23.9 | 7.1 |
| 3 | 380 | 1.5 | 19.7 | 4.9 | 1.0 | 5.0 | 2.5 | 0.2 | -12.3 | -24.2 | 5.9 |
| 3 | 380 | 3 | 6.7 | 0.7 | 0.5 | 5.0 | 2.3 | 0.1 | -10.7 | -24.5 | 6.0 |
| 3 | 380 | 4 | 5.1 | 0.3 | 0.3 | 5.4 | 2.4 | 0.1 | -10.8 | -24.6 | 5.5 |
| 4 | 380 | 0.25 | 42.6 | 10.8 | 3.1 | 4.8 | 2.3 | 0.2 | -13.8 | -23.7 | 18.3 |
| 4 | 380 | 0.75 | 44.8 | 11.6 | 3.7 | 4.9 | 2.8 | 0.2 | -12.7 | -23.8 | 9.0 |
| 4 | 380 | 1.5 | 26.8 | 5.6 | 1.9 | 5.4 | 2.8 | 0.2 | -13.5 | -23.7 | 6.5 |
| 4 | 380 | 4 | 3.7 | 0.4 | 0.2 | 5.2 | 1.9 | 0.1 | -10.7 | -24.9 | 5.3 |
| 5 | 540 | 0.25 | 20.1 | 2.6 | 1.8 | 4.3 | 2.1 | 0.2 | -11.7 | -24.3 | 22.0 |
| 5 | 540 | 0.75 | 26.7 | 8.0 | 1.3 | 4.9 | 2.7 | 0.2 | -13.1 | -24.2 | 10.0 |
| 5 | 540 | 1.5 | 11.8 | 1.3 | 0.7 | 5.3 | 2.3 | 0.1 | -12.9 | -24.3 | 6.8 |
| 5 | 540 | 3 | 6.1 | 0.7 | 0.4 | 4.9 | 2.0 | 0.1 | -10.0 | -24.5 | 5.3 |
| 5 | 540 | 4 | 2.0 | 0.2 | 0.1 | 8.6 | 2.3 | 0.2 | -17.1 | -25.1 | 3.4 |
| 6 | 540 | 0.25 | 12.1 | 1.6 | 1.1 | 3.2 | 2.4 | 0.1 | -12.3 | -24.0 | 10.4 |
| 6 | 540 | 0.75 | 24.1 | 6.1 | 1.5 | 5.8 | 2.3 | 0.2 | -14.2 | -24.1 | 6.6 |
| 6 | 540 | 1.5 | 18.4 | 6.3 | 0.7 | 5.1 | 2.4 | 0.2 | -12.0 | -24.3 | 4.2 |
| 6 | 540 | 3 | 7.4 | 1.1 | 0.4 | 5.3 | 2.4 | 0.1 | -12.0 | -24.8 | 3.9 |
| 7 | 540 | 0.25 | 23.2 | 6.5 | 1.4 | 4.4 | 2.6 | 0.2 | -13.0 | -23.8 | 4.9 |
| 7 | 540 | 0.75 | 21.6 | 4.4 | 1.7 | 5.0 | 2.3 | 0.2 | -13.1 | -23.8 | 4.8 |
| 7 | 540 | 1.5 | 21.9 | 3.3 | 1.5 | 5.6 | 2.5 | 0.2 | -13.3 | -24.2 | 4.4 |
| 7 | 540 | 3 | 10.6 | 0.8 | 0.8 | 5.0 | 2.2 | 0.1 | -11.5 | -25.0 | 5.5 |
| 7 | 540 | 4 | 3.8 | 0.2 | 0.3 | 7.1 | 3.9 | 0.1 | -15.9 | -24.6 | 3.9 |
| 8 | 540 | 0.25 | 34.4 | 10.4 | 2.0 | 4.3 | 2.7 | 0.2 | -12.4 | -23.9 | 20.4 |
| 8 | 540 | 0.75 | 31.4 | 10.0 | 1.9 | 5.5 | 3.2 | 0.2 | -12.2 | -24.1 | 9.0 |
| 8 | 540 | 1.5 | 24.1 | 5.5 | 1.6 | 5.4 | 3.0 | 0.2 | -12.8 | -24.0 | 7.4 |
| 8 | 540 | 3 | 9.1 | 1.7 | 0.4 | 4.9 | 2.5 | 0.1 | -10.8 | -20.7 | 5.5 |
| 8 | 540 | 4 | 3.0 | 0.2 | 0.2 | 6.8 | 3.6 | 0.1 | -14.5 | -24.7 | 4.8 |
| 9 | 760 | 0.25 | 24.6 | 6.0 | 1.9 | 4.8 | 2.5 | 0.2 | -12.6 | -23.7 | 29.8 |
| 9 | 760 | 0.75 | 19.0 | 4.8 | 1.3 | 5.7 | 2.9 | 0.2 | -13.2 | -23.9 | 11.6 |
| 9 | 760 | 1.5 | 15.4 | 1.8 | 1.1 | 5.4 | 2.6 | 0.2 | -13.0 | -23.7 | 6.7 |
| 9 | 760 | 3 | 8.8 | 1.1 | 0.5 | 5.3 | 2.7 | 0.1 | -11.4 | -24.6 | 4.8 |
| 9 | 760 | 4 | 2.3 | 0.2 | 0.3 | 5.0 | 3.2 | 0.1 | -11.1 | -24.6 | 5.0 |
| 10 | 760 | 0.25 | 42.4 | 15.7 | 2.3 | 4.4 | 2.3 | 0.2 | -13.2 | -23.8 | 16.6 |
| 10 | 760 | 0.75 | 28.5 | 7.7 | 1.7 | 5.3 | 3.2 | 0.2 | -13.6 | -22.0 | 7.4 |
| 10 | 760 | 1.5 | 17.2 | 4.0 | 1.0 | 5.0 | 2.7 | 0.2 | -11.8 | -24.0 | 5.8 |
| 10 | 760 | 3 | 7.1 | 0.3 | 0.5 | 5.0 | 2.3 | 0.1 | -9.6 | -23.2 | 8.1 |
| 10 | 760 | 4 | 2.7 | 0.2 | 0.2 | 8.4 | 4.2 | 0.2 | -17.1 | -24.9 | 4.5 |
| 11 | 760 | 0.25 | 26.8 | 3.2 | 2.5 | 4.6 | 2.9 | 0.2 | -12.3 | -18.0 | 23.0 |
| 11 | 760 | 0.75 | 20.2 | 4.0 | 1.5 | 4.3 | 2.7 | 0.2 | -11.7 | -13.1 | 8.5 |
| 11 | 760 | 1.5 | 23.2 | 5.0 | 1.5 | 5.2 | 3.4 | 0.2 | -12.5 | -24.0 | 6.9 |
| 11 | 760 | 3 | 11.4 | 1.0 | 0.6 | 5.1 | 2.7 | 0.1 | -12.3 | -24.2 | 5.6 |
| 11 | 760 | 4 | 4.1 | 0.1 | 0.3 | 6.6 | 2.7 | 0.1 | -14.3 | -24.8 | 4.6 |
| 12 | 760 | 0.25 | 27.8 | 4.5 | 2.5 | 4.3 | 2.7 | 0.2 | -12.6 | -23.6 | 14.5 |
| 12 | 760 | 0.75 | 22.4 | 8.4 | 1.2 | 5.2 | 3.1 | 0.2 | -12.7 | -21.0 | 5.5 |
| 12 | 760 | 1.5 | 19.9 | 2.4 | 1.6 | 5.5 | 2.9 | 0.2 | -13.4 | -24.1 | 5.3 |
| 12 | 760 | 3 | 12.0 | 1.1 | 0.9 | 4.7 | 2.6 | 0.1 | -12.9 | -20.8 | 5.4 |
| 12 | 760 | 4 | 3.3 | 0.2 | 0.2 | 4.9 | 2.1 | 0.1 | -10.1 | -24.5 | 4.4 |
| 13 | 1120 | 0.25 | 5.4 | 0.3 | 0.5 | 2.4 | 0.2 | 0.0 | 0.2 | -15.8 | 22.8 |
| 13 | 1120 | 0.75 | 1.7 | 0.2 | 0.2 | 2.0 | 0.1 | 0.0 | 0.9 | -15.8 | 44.0 |
| 13 | 1120 | 1.5 | 1.0 | 0.1 | 0.1 | 2.0 | 0.1 | 0.0 | 0.6 | -16.6 | 17.3 |
| 13 | 1120 | 3 | 0.4 | 0.0 | 0.0 | 2.1 | 0.1 | 0.0 | 1.2 | -22.9 | 12.4 |
| 13 | 1120 | 4 | 0.2 | 0.0 | 0.0 | 2.1 | 0.1 | 0.0 | 1.1 | -17.6 | 4.3 |
| 14 | 1120 | 0.25 | 4.7 | 0.3 | 0.5 | 2.5 | 0.1 | 0.0 | 0.9 | -20.0 | 22.4 |
| 14 | 1120 | 0.75 | 1.3 | 0.1 | 0.1 | 1.9 | 0.1 | 0.0 | 0.9 | -18.7 | 45.1 |
| 14 | 1120 | 1.5 | 1.0 | 0.1 | 0.1 | 1.8 | 0.1 | 0.0 | 1.1 | -18.2 | 17.3 |
| 15 | 1120 | 0.25 | 18.6 | 6.5 | 1.0 | 3.9 | 2.1 | 0.2 | -11.4 | -23.3 | 14.2 |
| 15 | 1120 | 0.75 | 25.4 | 8.7 | 1.5 | 5.2 | 2.9 | 0.2 | -13.0 | -23.7 | 6.1 |
| 15 | 1120 | 1.5 | 12.9 | 0.9 | 1.1 | 5.2 | 2.8 | 0.2 | -12.6 | -24.0 | 5.5 |
| 15 | 1120 | 3 | 4.4 | 0.3 | 0.3 | 5.1 | 2.3 | 0.1 | -10.2 | -24.3 | 5.5 |
| 15 | 1120 | 4 | 8.2 | 0.7 | 0.5 | 5.1 | 2.2 | 0.1 | -8.8 | -16.2 | 5.5 |
| 16 | 1120 | 0.25 | 24.3 | 6.5 | 1.5 | 5.2 | 3.1 | 0.2 | -12.5 | -24.0 | 5.0 |
| 16 | 1120 | 0.75 | 18.9 | 4.1 | 1.2 | 5.4 | 2.8 | 0.2 | -13.2 | -23.7 | 6.9 |
| 16 | 1120 | 1.5 | 15.9 | 3.2 | 1.0 | 5.3 | 2.5 | 0.2 | -12.0 | -24.1 | 7.0 |
| 16 | 1120 | 3 | 7.7 | 0.8 | 0.4 | 5.3 | 2.5 | 0.1 | -11.1 | -24.2 | 5.5 |
| 16 | 1120 | 4 | 5.0 | 0.3 | 0.3 | 5.2 | 2.4 | 0.1 | -9.8 | -24.1 | 4.8 |
| 17 | 3000 | 0.25 | 16.9 | 2.3 | 1.6 | 4.3 | 2.4 | 0.2 | -11.8 | -23.9 | 21.9 |
| 17 | 3000 | 0.75 | 27.2 | 7.0 | 1.8 | 5.0 | 2.8 | 0.2 | -12.6 | -23.9 | 9.1 |
| 17 | 3000 | 1.5 | 28.9 | 6.8 | 1.9 | 5.1 | 2.8 | 0.2 | -12.1 | -23.9 | 7.1 |
| 17 | 3000 | 3 | 7.9 | 0.7 | 0.5 | 5.1 | 2.5 | 0.1 | -11.2 | -23.4 | 7.3 |
| 17 | 3000 | 4 | 3.8 | 0.2 | 0.4 | 6.1 | 2.6 | 0.1 | -13.3 | -23.9 | 5.1 |
| 18 | 3000 | 0.25 | 20.4 | 3.9 | 1.4 | 4.6 | 2.5 | 0.2 | -13.6 | -23.1 | 10.4 |
| 18 | 3000 | 0.75 | 32.9 | 6.4 | 2.2 | 5.5 | 3.0 | 0.2 | -13.5 | -23.4 | 6.6 |
| 18 | 3000 | 1.5 | 16.6 | 2.0 | 1.0 | 5.1 | 2.7 | 0.2 | -12.0 | -23.8 | 5.7 |
| 18 | 3000 | 3 | 5.2 | 0.4 | 0.3 | 4.9 | 2.5 | 0.1 | -9.2 | -23.9 | 5.3 |
| 18 | 3000 | 4 | 1.8 | 0.1 | 0.2 | 9.5 | 3.5 | 0.2 | -18.3 | -24.8 | 1.7 |
| 19 | 3000 | 0.25 | 26.9 | 4.3 | 1.7 | 6.5 | 2.7 | 0.2 | -16.9 | -22.9 | 4.2 |
| 19 | 3000 | 0.75 | 20.4 | 2.5 | 1.7 | 5.2 | 3.0 | 0.2 | -12.1 | -23.8 | 5.6 |
| 19 | 3000 | 1.5 | 12.4 | 1.5 | 0.8 | 5.1 | 2.8 | 0.1 | -11.5 | -23.1 | 7.0 |
| 19 | 3000 | 3 | 6.4 | 0.5 | 0.5 | 5.6 | 2.2 | 0.1 | -11.2 | -21.2 | 5.5 |
| 19 | 3000 | 4 | 7.4 | 0.3 | 0.5 | 5.6 | 2.7 | 0.1 | -11.4 | -23.8 | 5.9 |
| 20 | 3000 | 0.25 | 25.5 | 6.8 | 1.6 | 5.1 | 3.6 | 0.2 | -13.9 | -22.4 | 7.6 |
| 20 | 3000 | 0.75 | 25.5 | 5.8 | 1.6 | 5.5 | 3.0 | 0.2 | -13.3 | -23.9 | 9.9 |
| 20 | 3000 | 1.5 | 10.3 | 1.1 | 0.7 | 4.5 | 2.4 | 0.1 | -11.0 | -24.2 | 6.6 |
| 20 | 3000 | 3 | 5.0 | 0.4 | 0.3 | 5.2 | 2.4 | 0.1 | -9.4 | -24.2 | 5.0 |
