# Supplementary material for: Impacts of Ocean Acidification on Sediment Processes in Shallow Waters of the Arctic Ocean
Source: PLoS One. 2014 Apr 9;9(4):e94068. doi: 10.1371/journal.pone.0094068 (PMC3981760; doi:10.1371/journal.pone.0094068)
Supplement: Table S2 — Correlation matrix associated with the PCA performed on the composition of the sediment at the end of the experiment (14 days) with respect to carbon and nitrogen (Ctot and Ntot), organic carbon (Corg), the isotopic composition of Ctot and Corg (δ13Ctot and δ13Corg) and organic nitrogen (δ15Norg), as well as the pigments concentrations (chlorophyll a , b and c ). Seawater targeted partial pressure of CO2 (pCO2 in μatm) and the depth of sampling (cm) in the sediment have been added as supplementary variables. The first two axes of the PCA represented 45 and 27% of the variance, respectively. All correlation values were tested for significance (Pearson correlation test performed on R, package Psych). Values in bold are associated with a p value below 0.01. (DOCX) [file pone.0094068.s002.docx]

|  | *p*CO_2_ | Depth | N_tot_ | δ^13^C_org_ | δ^13^C_tot_ | δ^15^N_org_ | C_org_ | C_tot_ | Chl *a* | Chl *b* | Chl *c* | Axis 1 | Axis 2 |
| --- | --- | --- | --- | --- | --- | --- | --- | --- | --- | --- | --- | --- | --- |
| *p*CO_2_ |  | -0.01 | -0.02 | 0.07 | -0.04 | -0.14 | 0.12 | 0.12 | -0.04 | -0.09 | -0.03 | 0.06 | 0.12 |
| Depth | -0.01 |  | **-0.74** | -0.15 | 0.17 | **-0.55** | -0.01 | **0.49** | **-0.84** | **-0.73** | **-0.83** | **0.83** | 0.3 |
| Ntot | -0.02 | **-0.74** |  | 0.13 | **-0.58** | 0.34 | 0.33 | 0.04 | **0.82** | **0.78** | **0.78** | **-0.93** | 0.21 |
| δ^13^C_org_ | 0.07 | -0.15 | 0.13 |  | 0.15 | 0.08 | -0.02 | -0.22 | 0.16 | 0.09 | 0.17 | -0.16 | -0.28 |
| δ^13^C_tot_ | -0.04 | 0.17 | **-0.58** | 0.15 |  | 0.03 | **-0.56** | **-0.66** | -0.24 | -0.23 | -0.22 | **0.44** | **-0.8** |
| δ^15^N_org_ | -0.14 | **-0.55** | **0.34** | 0.08 | 0.03 |  | -0.17 | **-0.44** | **0.44** | 0.37 | **0.51** | **-0.5** | **-0.46** |
| C_org_ | 0.12 | -0.01 | **0.33** | -0.02 | **-0.56** | -0.17 |  | **0.48** | 0.1 | 0.11 | 0.09 | -0.27 | **0.73** |
| C_tot_ | 0.12 | **0.49** | 0.04 | -0.22 | **-0.66** | **-0.44** | **0.48** |  | -0.35 | -0.28 | -0.36 | 0.18 | **0.91** |
| Chl *a* | -0.04 | **-0.84** | **0.82** | 0.16 | -0.24 | **0.44** | 0.1 | **-0.35** |  | **0.92** | **0.96** | **-0.94** | -0.19 |
| Chl *b* | -0.09 | **-0.73** | **0.78** | 0.09 | -0.23 | **0.37** | 0.11 | -0.28 | **0.92** |  | **0.8** | **-0.91** | -0.14 |
| Chl *c* | -0.03 | **-0.83** | **0.78** | 0.17 | -0.22 | **0.51** | 0.09 | **-0.36** | **0.96** | **0.8** |  | **-0.89** | -0.2 |
| Axis 1 | 0.06 | **0.83** | **-0.93** | -0.16 | **0.44** | **-0.5** | -0.27 | 0.18 | **-0.94** | **-0.91** | **-0.89** |  |  |
| Axis 2 | 0.12 | 0.3 | 0.21 | -0.28 | **-0.8** | **-0.46** | **0.73** | **0.91** | -0.19 | -0.14 | -0.2 |  |  |
