# Supplementary material for: Impacts of Ocean Acidification on Sediment Processes in Shallow Waters of the Arctic Ocean
Source: PLoS One. 2014 Apr 9;9(4):e94068. doi: 10.1371/journal.pone.0094068 (PMC3981760; doi:10.1371/journal.pone.0094068)
Supplement: Table S3 — Correlation matrix associated with the PCA performed on the sediment-water fluxes of total alkalinity (Δ A T), dissolved inorganic carbon (Δ C T), dissolved oxygen (ΔDO), ammonium (ΔNH4+), nitrite (ΔNO2−), nitrate (ΔNO3−), dissolved inorganic phosphate (ΔDIP) and silicate (ΔSi). Seawater temperature (Temp) and partial pressure of CO2 (pCO2) at which sediment cores were exposed during the 24 h incubations (average between initial and final sampling) as well as the time elapsed since the start of the experiment (in days) have been added as supplementary variables. The first two axes of the PCA represented 32 and 21% of the variance, respectively. All correlation values were tested for significance (Pearson correlation test performed using the R software, package Psych). Values in bold are associated with a p value below 0.01. (DOCX) [file pone.0094068.s003.docx]

|  | Time | Temp | *p*CO_2_ | Δ*A*_T_ | Δ*C*_T_ | ΔDO | ΔNH_4_^+^ | ΔNO_2_^-^ | ΔNO_3_^-^ | ΔDIP | ΔSi | Axis 1 | Axis 2 |
| --- | --- | --- | --- | --- | --- | --- | --- | --- | --- | --- | --- | --- | --- |
| Time |  | **0.57** | 0.00 | 0.03 | -0.09 | -0.13 | -0.04 | 0.02 | 0.10 | -0.01 | **0.49** | 0 | -0.28 |
| Temp | **0.57** |  | -0.04 | -0.12 | -0.15 | -0.05 | -0.06 | 0.20 | -0.06 | -0.13 | 0.10 | 0.12 | 0.04 |
| *p*CO_2_ | 0.00 | -0.04 |  | **0.73** | **0.87** | 0.10 | **-0.38** | **-0.57** | -0.19 | -0.14 | 0.28 | **-0.85** | -0.17 |
| Δ*A*_T_ | 0.03 | -0.12 | **0.73** |  | **0.73** | 0.13 | -0.28 | -0.31 | -0.04 | -0.12 | 0.27 | **-0.75** | -0.3 |
| Δ*C*_T_ | -0.09 | -0.15 | **0.87** | **0.73** |  | -0.01 | -0.32 | **-0.61** | -0.14 | -0.06 | **0.33** | **-0.88** | -0.28 |
| ΔDO | -0.13 | -0.05 | 0.10 | 0.13 | -0.01 |  |  | 0.04 | 0.07 | -0.07 | -0.08 | -0.1 | 0.12 |
| ΔNH_4_^+^ | -0.04 | -0.06 | **-0.38** | -0.28 | **-0.32** | -0.17 |  | **0.56** | 0.21 | 0.14 | 0.26 | **0.64** | **-0.46** |
| ΔNO_2_^-^ | 0.02 | 0.20 | **-0.57** | **-0.31** | **-0.61** | 0.04 | **0.56** |  | 0.29 | 0.02 | -0.14 | **0.8** | -0.1 |
| ΔNO_3_^-^ | 0.10 | -0.06 | -0.19 | -0.04 | -0.14 | 0.07 | 0.21 | **0.29** |  | 0.11 | **0.40** | 0.3 | **-0.66** |
| ΔDIP | -0.01 | -0.13 | -0.14 | -0.12 | -0.06 | -0.07 | 0.14 | 0.02 | 0.11 |  | 0.13 | 0.17 | -0.31 |
| ΔSi | **0.49** | 0.10 | 0.28 | 0.27 | **0.33** | -0.08 | **0.26** | -0.14 | **0.40** | 0.13 |  | -0.19 | **-0.87** |
| Axis 1 | 0 | 0.12 | **-0.85** | **-0.75** | **-0.88** | -0.1 | **0.64** | **0.8** | **0.3** | 0.17 | -0.19 |  |  |
| Axis2 | -0.28 | 0.04 | -0.17 | **-0.3** | -0.28 | 0.12 | **-0.46** | -0.1 | **-0.66** | **-0.31** | **-0.87** |  |  |
